# The Impact of Treatment Setting on Periurethral Bulking

**DOI:** 10.1007/s00192-025-06236-5

**Published:** 2025-09-20

**Authors:** Erin E. Mowers, Mary F. Ackenbom, Lauren E. Giugale, Amanda M. Artsen

**Affiliations:** 1 Division of Urogynecology and Reconstructive Pelvic Surgery, Department of Obstetrics, Gynecology, and Reproductive Sciences, UPMC Magee-Womens Hospital, Pittsburgh, PA, USA; 2 Magee-Womens Research Institute, Pittsburgh, PA, USA; 3 Division of Urogynecology and Reproductive Pelvic Surgery, Department of Obstetrics & Gynecology, University of Illinois College of Medicine Peoria, Peoria, IL, USA; 4 Division of Urogynecology and Reconstructive Pelvic Surgery, Department of Obstetrics and Gynecology, University of Michigan, Ann Arbor, MI, USA

**Keywords:** Bulkamid, Cystoscopy, Treatment outcome, Urinary incontinence, Urinary stress incontinence

## Abstract

**Introduction and Hypothesis:**

Whether treatment setting impacts periurethral bulking efficacy is unknown. We hypothesized that periurethral bulking treatment failure rates would be greater for office versus operating room procedures.

**Methods:**

A retrospective cohort study including 113 patients undergoing polyacrylamide hydrogel periurethral bulking at a single academic medical center between 1 September 2022 and 30 March 2024 was carried out. Demographic and clinical variables, procedural details, patient satisfaction, and retreatment rates were abstracted. Primary outcomes included 6-month treatment failure, defined as no improvement following treatment, decision for additional therapy, or aborted procedure. Secondary outcomes included 12-month treatment failure and 6- and 12-month retreatment rates. Chi-squared, Fisher’s exact, Mann–Whitney *U*, and *t* tests were applied, and logistic regression was used to identify factors associated with treatment failure.

**Results:**

The 6- and 12-month treatment failure rates in the operating room versus office cohorts were 32.4% vs 51.3% (*p* = 0.05) and 48.1% vs 50.0% (*p* = 0.87) respectively. Retreatment rates and modality did not differ. Approximately 15% of office cases but no operating room cases were unable to be completed as planned (*p* < 0.01). Office setting was associated with 3.23 increased odds of 6-month treatment failure (*p* < 0.05).

**Conclusions:**

Periurethral bulking in the office may be more likely to result in early treatment failure than bulking performed in the operating room, but any benefit appears short lived, with similar 12-month treatment failure and retreatment rates. Our findings, together with consideration of individual patient characteristics and resource optimization, can guide decisions regarding the appropriate treatment setting for periurethral bulking.

## Introduction

Stress urinary incontinence (SUI) is the involuntary leakage of urine that occurs when intra-abdominal pressure exceeds urethral pressure, as may occur during coughing, laughing, and sneezing [1]. Half of all women experience SUI in their lifetime, and 14% undergo surgery [1, 2]. Treatment ranges from conservative therapies such as physical therapy or incontinence pessaries to surgical treatment. Of the surgical options, periurethral bulking (PUB), in which a cystourethroscope is used to inject bulking material into the urethral submucosa, is the least invasive and may be particularly useful in women with an elevated surgical risk or future childbearing plans [1].

Polyacrylamide hydrogel (PAH; Axonics, Irvine, CA, USA), 97% water by volume suspended in nontoxic polyacrylamide, creates a bulking effect owing to entry of host cells into the gel and the formation of fibrous networks linking the gel to urethral tissue [3]. Although response rates appear similar to those of other bulking agents, PAH may exhibit a superior safety profile, with lower rates of serious adverse events [3, 4]. PAH-PUB, hereafter referred to simply as PUB, may be used with good success for primary or refractory SUI [5, 6]. Approximately two-thirds of patients report improvement in symptoms following PUB, and one-third achieve complete cure [3, 7–11]. Responses are durable, with up to 67% of patients reporting cure or improvement 7 years after injection [4, 8, 11]. Complications due to PUB are uncommon but can include transient urinary retention and urinary tract infection (UTI) [3, 5, 7, 11, 12].

Several studies have demonstrated that PUB may be successfully and safely performed in the outpatient setting, but whether this more convenient and lower-cost approach impacts efficacy is not well understood [13, 14]. One potential downside to PUB performed in the office is the potential for greater patient discomfort with only local anesthesia, which could potentially lead to suboptimal injection locations or volumes. Therefore, we aimed to assess whether a difference in failure rates exists following initial PUB performed in the operating room (OR) compared with the office setting, hypothesizing that failure rates would be higher for office PUB.

## Materials and Methods

### Patient Population

This exploratory study using a retrospective cohort design included data from all female patients who underwent primary PUB with PAH in the office or OR in the Division of Urogynecology at a large US academic medical center between 1 September 2022 and 30 March 2024. Institutional Review Board study exemption was obtained (IRB#: 24030014). Study design adhered to Strengthening the Reporting of Observational Studies in Epidemiology guidelines [15]. Surgical calendars and office schedules for nine surgeons at two sites were reviewed to identify all patients undergoing primary PUB. Non-PAH PUB cases were excluded, as were patients with prior PUB with PAH and those without at least one clinical follow-up contact after a successfully completed PUB procedure.

### Procedures

Patient eligibility for PUB was determined at the discretion of the clinician after history and physical examination; no specific objective criteria were required for inclusion in this study. Decisions to pursue PAH-PUB were reached through joint decision making between the patient and the surgeon. At our institution, surgeons select the PUB procedure location individually for each patient based on patient and surgeon preference, as well as the timing and availability of appointments and surgical blocks. PUB in the office is performed within 30 min following the application of 2% viscous lidocaine gel to the urethra; no periurethral block, anxiolytics, or other anesthesia is provided. Antibiotic prophylaxis is not routinely provided for office PUB but may be administered for concurrent onabotulinumtoxinA injections or at provider discretion. OR procedures are performed under intravenous (IV) sedation unless additional surgical procedures requiring general anesthesia are performed, and IV antibiotic prophylaxis with a third-generation cephalosporin is provided. For OR procedures, viscous lidocaine was applied at the surgeon’s discretion, and periurethral blocks were not performed. In either setting, the amount of PAH is titrated to achieve adequate urethral coaptation following the manufacturer’s guidelines. A Valsalva stress test (VST) following PUB may be performed in either the office or OR (after the patient is awoken from sedation) according to the surgeon’s preference, with a positive VST prompting the application of additional PAH. The same group of surgeons performed office procedures as did OR procedures, and all surgeons had a minimum of 6 months of PAH experience and a minimum of 3 years of PUB experience. Deviations from this protocol occurred at the discretion of the surgeon and are noted.

### Clinical Variables

Electronic medical records (EMRs) were reviewed to obtain demographic and clinical variables, including obstetric history, comorbidities, medications, prior SUI treatment (physical therapy, incontinence pessary, or SUI surgery), SUI workup (including confirmation of SUI and post-void residual [PVR]), procedural details (type of anesthesia, antibiotic prophylaxis, concurrent procedures, injection volume, etc.), and postoperative urinary retention and UTI. All post-procedure encounters within the urogynecology division during the 6- and 12-month follow-up timeframe were reviewed, including operative encounters, clinical visits, telephone calls, and electronic messaging documentation. The reported subjective satisfaction as documented by the clinician during these encounters was classified as worse, unchanged, improved, or cured. Encounters were also reviewed to identify patients who requested additional post-procedure treatment for ongoing SUI symptoms, including repeat injection (PAH or other), SUI surgery, incontinence pessary, or physical therapy; pessary or physical therapy for non-SUI indications was not considered retreatment. Any patient undergoing retreatment was analyzed only in their original cohort, regardless of the retreatment modality and setting.

### Outcomes

Our study was motivated by the question a clinician may ask herself when a decision is made to pursue PUB—is this patient better served by an office or OR procedure? Thus, we selected a patient-centered definition of treatment failure that emphasized the outcome of a single PUB attempt. The focus on a single PUB attempt would allow us to detect a difference between treatment settings in the event that, for example, resolution of symptoms required only one injection in one setting but tended to require two injections if performed in the other setting. The primary outcome was 6-month composite treatment failure. Treatment failure was defined as a composite of:

1. Unchanged or worsened SUI symptoms
2. Retreatment for ongoing SUI symptoms documented as of the last clinical encounter within the follow-up period (including repeat PUB, pessary, physical therapy, midurethral sling, etc.)
3. Need to abort the PUB procedure owing to patient tolerance or anatomical challenges

Patients who initially reported worsening incontinence symptoms but subsequently experienced improvement following UTI treatment were not considered treatment failures unless they met one of the other above criteria. Once a patient was determined to have a treatment failure, they were considered to have a treatment failure for all subsequent timepoints. Secondary outcomes included 12-month composite treatment failure and 6- and 12-month retreatment rates. Retreatment was defined as a decision made to proceed with additional therapy within the follow-up period, irrespective of the date for which the retreatment was ultimately scheduled.

### Sensitivity Analysis

Sensitivity analyses were performed using four alternate analyses of composite failure. In the first, patients who did not have at least one postoperative encounter (office or telehealth visit, phone call, or electronic message) with the urogynecology division following PUB were considered to have treatment success. In the second, treatment failure was assumed for patients who did not have at least one post-procedure encounter. In the third, patients undergoing any procedure in addition to PUB (e.g., prolapse repair, onabotulinumtoxinA) were excluded from the analysis, and treatment failure was defined as in the original analysis. Finally, in the fourth, patients whose procedure was aborted were excluded, and only completed procedures were analyzed; treatment failure was defined as unchanged or worsened SUI symptoms, or retreatment for SUI symptoms.

### Statistical Analysis

To examine associations, Chi-squared and Fisher’s exact tests were used for categorical variables, and Mann–Whitney *U* and *t* tests were used for continuous variables as appropriate. Assuming a 6-month composite treatment failure of 30% for OR PUB [4, 6–10] and a two-fold increase in failure rates with office PUB, we calculated that 60 patients in the OR group and 30 patients in the office group would be needed to achieve 80% power at α = 0.05. Given the lack of published clinical data regarding success rates for PUB performed in the office, we based our twofold increase in failure rates on our clinical observations for this exploratory study. Univariable and multivariable logistic regression were performed to identify factors associated with PUB failure and to control for confounders. Candidate variables with *p* < 0.2 on univariable analysis were used in the final modeling, with multivariable regression fitted with backward elimination. Concurrent prolapse surgery and type of anesthesia were excluded as candidates owing to collinearity with treatment setting. All statistical analyses were conducted using Stata/SE 18.0 (StataCorp, College Station, TX, USA).

## Results

During the study period, 74 patients underwent PUB in the OR, and 39 patients underwent PUB in the office (Table 1). The majority of patients were white (99.1%, *n* = 112) and non-Latinx (93.8%, *n* = 106). The mean age was 63.1 years (standard deviation [SD] = 14.7), and the mean body mass index (BMI) was 31.3 kg/m^2^ (SD = 8.4). There were no differences in race, ethnicity, age, BMI, insurance status, prolapse stage, obstetric history, medications, and comorbidities (with the exception of fibromyalgia) between the groups (Table 1, all *p* > 0.05 with the exception of fibromyalgia). Similarly, rates and types of prior SUI therapy, rates of prior hysterectomy or SUI surgery, diagnosis of SUI, PVR, and VST volume were similar in the groups (Table 1, all *p* > 0.05). Despite the anticipated procedural differences inherent to the institutional OR and office PUB protocols (e.g., type of anesthesia), no differences were observed in the volume of PAH injected, but rates of aborted procedures were higher in the office cohort (Table 2). Of the 9 surgeons performing PUB, 1 performed only OR procedures (*n* = 13), and 1 performed only office procedures (*n* = 2). None of the remaining seven surgeons who performed both OR and office procedures exhibited a statistically significant preference for either procedure setting (all *p* > 0.05).

With respect to our primary outcomes, the 6-month treatment failure incidence was 32.4% (*n* = 24) in the OR cohort and 51.3% (*n* = 20) in the office cohort; this difference was just above the threshold for statistical significance (*p* = 0.05; Table 3). None of our secondary outcomes differed statistically between the cohorts with the exception of an increased incidence of post-procedure urinary retention in the OR cohort, but this difference lost significance when restricting the analysis to PUB-only procedures (Table 3). At 12 months, the differences in the treatment failure rates between the OR and office cohorts was minimal (48.1% [*n* = 25] vs 50.0% [*n* = 15] respectively; *p* = 0.87).

Sensitivity analyses were performed to assess the robustness of the results using three alternative inclusion/exclusion criteria (Table 4). Notably, whether patients who were lost to follow-up were assumed to have treatment success or failure impacted whether the observed 6-month treatment failure rates were statistically significant, as did restricting our analysis to PUB-only procedures. Excluding patients whose procedure was aborted and analyzing only completed procedures resulted in no significant difference in failure rates. Treatment failure rates at 12 months did not differ between the OR and office cohorts irrespective of the analysis performed (data not shown). When patients lost to follow-up were compared with patients with at least one point of follow-up contact, patients not identifying as being of white race made up a larger proportion of the group that was lost to follow-up than the study cohorts (*n* = 3 [13.6%] vs *n* = 1 [0.9%] respectively; *p* < 0.05). There were no other differences between patients lost to follow-up and those included in the study with respect to other demographic and clinical characteristics, prior SUI therapy, SUI evaluation, or procedural characteristics (data not shown).

Univariable and multivariable analyses were conducted to identify factors associated with 6-month treatment failure. After univariable analysis of all demographic, clinical, and procedural characteristics, prolapse stage, use of overactive bladder (OAB) medication, use of opioid medication, hypertension, chronic obstructive pulmonary disease (COPD), pelvic pain composite (pelvic pain, levator spasm, fibromyalgia, or female bladder pain syndrome), and office setting met the criteria for inclusion in the multivariable model (Table 5, all *p* < 0.2). The final model identified use of OAB medication, COPD, composite pain, and office setting as factors significantly associated with 6-month treatment failure (Table 5, all *p* < 0.05).

## Discussion

Our retrospective cohort pilot study of 113 patients examined the association between treatment setting and treatment failure following PUB with PAH. We observed composite 6-month treatment failure in 32.4% of patients undergoing PUB in the OR compared with 51.3% of patients following office PUB, a difference that was just above our threshold for significance. However, on multivariable analysis, the office setting was significantly associated with 6-month treatment failure. By 12 months, approximately half of all patients experienced treatment failure irrespective of their original treatment setting. Retreatment rates did not differ at either timepoint. Although all OR procedures were successfully completed, 15% of office procedures had to be aborted owing to patient tolerance or anatomical challenges.

The 6- and 12-month success rates in our study are notably lower than the reported two-thirds efficacy reported in other studies at up to 7 years [3, 7–11]. However, the majority of studies of PAB efficacy using PAH consider two or three total injections with subsequent satisfactory benefit to reflect successful treatment, whereas in our study, the need for any repeat injections was defined as treatment failure [8, 10–12, 16]. The reason for this endpoint difference lies in the fact that our study was not intended to assess efficacy per se but to determine whether treatment setting alters efficacy. The need for additional “touch-up” reinjections to improve symptoms is common for PUB, occurring in approximately 25–50% of patients, and PAH reinjection may be more common than reinjection with other bulking agents [4, 8, 10, 12, 17]. With repeated PUB, treatment efficacy may improve to as much as 85% [17]. In our study, one-fifth of patients elected to undergo subsequent PUB, and it is possible, based on these reported success rates of reinjection, that many of these patients achieved satisfactory results thereafter.

We found that the rates of post-procedure urinary retention were significantly higher in the OR cohort than in the office cohort (25.0% [*n* = 18] vs 2.6% (*n* = 1) respectively; *p* < 0.01; Table 3). This difference appears to be largely related to concurrent prolapse procedures and the use of anesthesia in the OR cohort; when the analysis was restricted to patients who underwent only PUB procedures without other prolapse or incontinence procedures, the differences in the rates of urinary retention between the cohorts no longer reached statistical significance (15.9% [*n* = 7] vs 3.0% [*n* = 1] respectively; *p* = 0.13). Nonetheless, the trend toward increased retention in the OR cohort is consistent with the trend toward decreased composite failure in the OR cohort; more efficacious bulking would be expected to increase the likelihood of urinary retention. However, our study was not powered to detect differences in urinary retention, and neither the difference in retention nor the difference in composite failure was significant. Larger and appropriately powered studies will be required to explore this possible relationship further.

Periurethral bulking may be used as a primary treatment for SUI or as a salvage treatment in those with persistent SUI following other therapies [5–7, 11, 16]. Similarly, nearly one-fifth of patients undergo a subsequent incontinence procedure following PUB with PAH, consistent with our finding that approximately 12% of patients elected to undergo an incontinence surgery during the 6-month follow-up period [11]. Previous studies have identified age > 60 years, Incontinence Impact Questionnaire (IIQ7) scores, number of daily SUI episodes, and prior incontinence surgery as associated with PUB success [18, 19]. Notably, maximal urethral closing pressures and urethral hypermobility have not been associated with the efficacy of PUB [7, 20, 21]. On our final multivariable analysis, use of OAB medications, COPD, and composite pain were significantly associated with 6-month treatment failure.

Intriguingly, an office setting was also significantly associated with threefold increased odds of 6-month treatment failure on multivariable analysis, despite the lack of statistical difference in 6-month failure rates between our two cohorts. The reasons for this are likely multifactorial and are influenced by the sample size. Although we did satisfy our predetermined power calculation for 6-month outcomes, it is noteworthy that this calculation was based on a fairly large twofold difference threshold in treatment failure rates based on our initial clinical observations. Furthermore, our sensitivity analysis indicated that the treatment outcomes of the patients who were lost to follow-up impacted whether the observed 6-month difference in failure rates was significant, with a statistically significant difference observed if all patients lost-to-follow-up were treatment failures but a nonsignificant difference if they were treatment successes. Finally, although our cohorts were well-matched overall (Table 1), procedure setting was selected individually for each patient based on patient and surgeon preference as well as the timing and availability of appointments and surgical blocks, and selection bias may have led to inherent differences between the cohorts that were not captured in our study variables and thus may have obscured the association of treatment failure with procedure setting. Our multivariable analysis provides some protection against these influences to uncover the role of treatment setting in 6-month PUB failure rates. However, any definitive conclusions regarding the impact of treatment setting on PUB failure rates will require additional randomized controlled trials with larger sample sizes to better control for unmeasured differences between the cohorts.

Because our aim was to assess whether treatment setting alters efficacy, our definition of composite treatment failure included aborted procedures. None of these aborted procedures, all of which occurred in the office, represented equipment failure but rather were the result of patient tolerance or insufficient urethral diameter, despite the surgeon’s prior evaluation that an office procedure was feasible. We feel that these procedures represent treatment failures in the practical sense because the patient did not receive any of the anticipated treatment benefit. However, it is worth noting that if our analysis is limited only to procedures that could be successfully completed (i.e., if aborted procedures are excluded), the differences in 6- and 12-month treatment failure rates are blunted and do not reach statistical significance. Thus, the biggest downside to PUB in the office compared with the OR may be the increased risk of an aborted procedure, which represents both a delay in symptom treatment and a potential financial burden on both the patient and the surgeon. Careful patient selection may overcome this concern.

As a retrospective analysis, our pilot study has some limitations in addition to those above. Diagnosis of SUI and patient evaluation as a candidate for PUB was performed at the discretion of the surgeon. For our office procedures, 2% viscous lidocaine was the primary form of analgesia, and the addition of other forms of analgesia, including periurethral blocks or the use of anxiolytics, may further modulate the impact of treatment setting on PUB efficacy and should be considered when evaluating the generalizability of our findings. Further studies exploring the ideal analgesic setup for office procedures that considers treatment efficacy, patient experience, feasibility, costs, and resource allocation will continue to be important components of any discussion regarding treatment setting. Post-procedure symptom assessment was not standardized but was based on the documented effect in the EMR, which may have influenced our interpretation of treatment outcomes. Follow-up was similarly nonstandardized, and 12 patients (14.6%) in the OR cohort and 10 patients (20.4%) in the office cohort did not follow up after PUB and were not included in the primary analysis, although only race was found to differ statistically between patients who did and those who did not follow up. However, this difference is unlikely to be clinically relevant and reflects 1 patient of unknown race among those who did follow up versus 1 patient of Black race and 2 patients of unknown race among those who did not follow up (data not shown). Future prospective studies or randomized controlled trials including standardized symptom assessment (e.g., ICIQ-UI) and planned follow-up visits with sufficient power to detect smaller but clinically meaningful differences in treatment outcomes would help to address these remaining questions. Despite these limitations, our study is to our knowledge the first to examine the impact of treatment setting on PUB outcomes. We selected a practical and patient-focused definition of composite treatment failure, including any procedures that did not improve patient symptoms and those that required additional intervention (either because of insufficient improvement or an inability to perform the procedure as planned).

Although the present study represents only a preliminary exploration of the impact of treatment setting on PUB, our data suggest that PUB with PAH may be more likely to result in early (6-month) treatment failure when performed in the office, but any potential benefit of OR bulking over office bulking appears to be short lived, with similar 12-month treatment failure rates and similar retreatment rates at both time points. The increased risk of an aborted procedure when PUB is attempted in the office may drive the early differences in treatment failure, potentially highlighting the importance of careful patient evaluation prior to proceeding with an office procedure. This risk, combined with the finding that patients with COPD and pelvic pain conditions and those using OAB medications may be more likely to experience treatment failure, should be incorporated into the counseling and decision-making process for PUB. Our findings should prompt future randomized controlled trials with larger sample sizes to explore how treatment failure rates in different procedural settings, together with consideration of individual patient characteristics and resource optimization, can help to guide decisions regarding the appropriate treatment and setting for patients with SUI.

## Figures and Tables

**Table 1:** Baseline patient characteristics

	OR (*N* = 74)		Office (*N* = 39)		*p* value
	*n*	%	*n*	%	
Race					> 0.99
White	73	98.7	39	100.0	
Unknown	1	1.4	0	0.0	
Ethnicity					0.46
Non-Latinx	70	94.6	36	92.3	
Latinx	0	0.0	1	2.6	
Unknown	4	5.4	2	5.1	
Insurance					0.44
Private	34	46.0	13	33.3	
Medicare	32	43.2	23	60.0	
Medicaid	6	8.1	2	5.1	
None	2	2.7	1	2.6	
Age, years	62 (15.2)		65 (13.6)		0.23
BMI^a^, kg/m^2^	31.5 (8.4)		30.9 (8.4)		0.75
Prolapse					0.47
Stage 0 or 1	57	77.0	34	87.2	
Stage 2	13	17.6	3	7.7	
Stage 3	3	4.1	2	5.1	
Stage 4	1	1.4	0	0	
Obstetric^b^					
Gravida	2 (2–3)		3 (2–4)		0.17
Para	2 (1–3)		2 (1–3)		0.67
Medication					
Anticoagulant	4	5.4	1	2.6	0.66
OAB medication	23	31.1	18	46.2	0.11
Opioid	5	6.8	1	2.6	0.35
Non-opioid analgesic	25	33.8	10	25.5	0.37
Comorbidity					
Diabetes	14	18.9	10	25.6	0.41
Hypertension	41	55.4	22	56.4	0.92
OSA	1	14.9	8	20.5	0.45
COPD	5	6.8	3	7.7	> 0.99
Fibromyalgia	10	13.5	0	0.0	< *0.05*
MUI	45	60.8	24	61.5	0.94
Recurrent UTI	13	17.6	3	7.7	0.26
FBPS	3	4.1	0	0.0	0.55
Pelvic pain or levator spasm	18	24.3	4	10.3	0.09
Prior hysterectomy	31	41.9	18	46.2	0.66
Prior SUI therapy					
Any	46	62.2	18	46.2	0.10
Physical therapy	26	35.1	10	25.6	0.30
Incontinence pessary	13	17.6	4	10.3	0.41
Surgery					0.48
Burch	3	4.1	0	0.0	
Transobturator sling	5	6.8	3	7.7	
Retropubic sling	10	13.5	4	10.3	
Macroplastique	1	1.4	0	0.0	
Coaptite	1	1.4	0	0.0	
Other	2	2.7	0	0.0	
Multiple	0	0.0	2	5.1	
Evaluation					
Documentation of SUI					0.75
History only	9	12.2	5	12.8	
Valsalva stress test	36	48.7	23	59.0	
Cystometrics	7	9.5	4	10.3	
UDS	12	16.2	4	10.3	
Other^c^	10	13.5	3	7.7	
PVR^d^, ml	30 (10–50)		20 (5–55)		0.33
Stress test volume^e^, ml	110 (25–250)		75 (5–220)		0.18

Categorical variables are presented as *n*, with corresponding percentages as indicated. Continuous variables are presented as median (interquartile range) if non-normally distributed and as mean (standard deviation) if normally distributed

*BMI* body mass index, *COPD* chronic obstructive pulmonary disorder, *FBPS* female bladder pain syndrome, *MUI* mixed urinary incontinence, *OSA* obstructive sleep apnea, *OR* operating room, *OAB* overactive bladder, *PVR* post-void residual, *SUI* stress urinary incontinence, *UTI* urinary tract infection

Statistically significant values are indicated in italics

a OR: *n* = 73, office: *n* = 39

b OR: *n* = 71, office: *n* = 37

c Includes patients diagnosed with SUI based on symptoms in the setting of prior SUI therapy

d OR: *n* = 62, office: *n* = 35

e OR: *n* = 51, office: *n* = 29

**Table 2:** Characteristics of the periurethral bulking procedure

	OR, *N* = 74		Office, *N* = 39		*p* value
	*n*	%	*n*	%	
Anesthesia type					< *0.001*
Lidocaine gel	2	2.7	39	100.0	
Sedation	60	81.1	0	0.0	
General	12	16.2	0		
Antibiotic prophylaxis					*< 0.001*
Cefoxitin	59	82.0	0	0.0	
Cefazolin	10	13.9	0	0.0	
Macrobid	0	0.0	8	20.5	
Other	1	1.4	5	12.8	
None	2	2.8	26	66.7	
Injection volume^a^, ml	2 (2–2)		2 (1.7–2)		0.08
VST documented^a^	9	12.2	20	58.8	*< 0.001*
Procedure aborted^b^	0	0.0	6	15.4	*< 0.01*
Concurrent procedures					
OnabotulinumtoxinA	13	17.6	6	15.4	0.77
Pelvic floor injection	3	4.1	0	0.0	0.55
Prolapse surgery	7	9.5	0	0.0	0.09
Other surgery^c^	13	17.6	0	0.0	*< 0.01*

Categorical variables are presented as *n* (%). Injection volume is presented as median (interquartile range)

*OR* operating room, *VST* Valsalva stress test

Statistically significant values are indicated in italics

a OR: *n* = 74, office: *n* = 34

b Includes procedures aborted owing to patient tolerance (*n* = 2) or inability of the urethra to accommodate the cystourethroscope (*n* = 4); there were no cases of equipment failure

c Includes endometriosis excision, mesh removal, dilation and curettage, etc.

**Table 3:** Primary and secondary outcomes following periurethral bulking

	OR, *N* = 74		Office, *N* = 39		*p* value
	n	%	n	%	
6-month outcomes					
Treatment failure	24	32.4	20	51.3	0.05
Retreatment	17	23.0	13	33.3	0.24
12-month outcomes^a^					
Treatment failure	25	48.1	15	50.0	0.87
Retreatment	18	34.6	9	30.0	0.67
Post-procedure urinary retention					
All procedures^b^	18	25.0	1	2.6	< *0.01*
PUB-only procedures^c^	7	15.9	1	3.0	0.13
Post-procedure UTI					
All procedures	12	16.2	5	12.8	0.63
PUB-only procedures^c^	3	6.8	4	12.1	0.45
Retreatment selected^d^					
Urethral bulking	15	20.3	6	15.4	0.53
Physical therapy	2	2.7	4	10.3	0.18
Pessary	1	1.4	0	0	> 0.99
Surgery	9	12.2	5	12.8	0.92

Patients undergoing retreatment are presented only in their original cohorts; there are no crossovers

*OR* operating room, *PUB* periurethral bulking, *UTI* urinary tract infection

Statistically significant values are indicated in italics

a OR: *n* = 52, office: *n* = 30

b OR: *n* = 72, office: *n* = 39

c OR: *n* = 44, office: *n* = 33

d Some participants elected for more than one type of retreatment

**Table 4:** Sensitivity analysis for 6-month periurethral bulking treatment failure rates

	OR		Office		*p* value
	*n*	%	*n*	%	
Original analysis^a^	24	32.4	20	51.3	0.05
Alternative 1^b^: loss to follow-up assumed success	24	27.9	20	40.8	0.12
Alternative 2^b^: loss to follow-up assumed failure	36	41.9	30	61.2	*< 0.05*
Alternative 3^c^: PUB-only procedures	15	34.1	19	57.6	*< 0.05*
Alternative 4^d^: aborted procedures excluded	24	32.4	14	42.4	0.32

Lost to follow-up was defined as no follow-up contact (office or telehealth visit, telephone call, or electronic messaging) after the PUB procedure

Statistically significant values are presented in italics

*OR* operating room, *PUB* periurethral bulking

a OR, *n* = 74; office, *n* = 39

b OR, *n* = 86; office, *n* = 49

c OR, *n* = 44; office, *n* = 33

d OR, *n* = 74; office, *n* = 33

**Table 5:** Factors associated with 6-month treatment failure

	Unadjusted odds ratio (95% CI)	*p* value	Adjusted odds ratio (95% CI)	*p* value
Prolapse stage	0.66 (0.41–1.05)	0.08	0.60 (0.36–1.01)	0.05
OAB medication	2.63 (1.19–5.81)	*< 0.05*	2.59 (1.03–6.49)	< *0.05*
Opioid medication	3.35 (0.59–12.12)	0.17	4.71 (0.61–36.59)	0.14
Hypertension	0.5 (0.23–1.09)	0.08	0.42 (0.17–1.04)	0.06
COPD	5.29 (1.02–27.52)	*< 0.05*	2.08 (1.13–54.46)	*< 0.05*
Pelvic pain composite^a^	2.46 (1.04–5.82)	*< 0.05*	4.05 (1.40–11.76)	*< 0.05*
Office setting	2.19 (0.99–4.85)	0.05	3.23 (1.20–8.71)	*< 0.05*

Results are presented as odds ratios (95% CI). Variables with *p* < 0.2 on univariable analysis were candidate variables in multivariable analysis

Significant values are shown in italics

*CI* confidence interval, *OAB* overactive bladder, *COPD* chronic obstructive pulmonary disorder

a Pelvic pain composite includes pelvic pain/levator spasm, fibromyalgia, and female bladder pain syndrome

## Data Availability

The data that support the findings of this study are available from the corresponding author, E.E.M., upon reasonable request.
